# Discovery of an Abundance of Biosynthetic Gene Clusters in Shark Bay Microbial Mats

**DOI:** 10.3389/fmicb.2020.01950

**Published:** 2020-08-21

**Authors:** Ray Chen, Hon Lun Wong, Gareth S. Kindler, Fraser Iain MacLeod, Nicole Benaud, Belinda C. Ferrari, Brendan P. Burns

**Affiliations:** ^1^School of Biotechnology and Biomolecular Sciences, University of New South Wales, Sydney, NSW, Australia; ^2^Australian Centre for Astrobiology, University of New South Wales, Sydney, NSW, Australia

**Keywords:** biosynthetic gene cluster, microbial mat, secondary metabolite, natural product, genome mining, metagenomics

## Abstract

Microbial mats are geobiological multilayered ecosystems that have significant evolutionary value in understanding the evolution of early life on Earth. Shark Bay, Australia has some of the best examples of modern microbial mats thriving under harsh conditions of high temperatures, salinity, desiccation, and ultraviolet (UV) radiation. Microorganisms living in extreme ecosystems are thought to potentially encode for secondary metabolites as a survival strategy. Many secondary metabolites are natural products encoded by a grouping of genes known as biosynthetic gene clusters (BGCs). Natural products have diverse chemical structures and functions which provide competitive advantages for microorganisms and can also have biotechnology applications. In the present study, the diversity of BGC were described in detail for the first time from Shark Bay microbial mats. A total of 1477 BGCs were detected in metagenomic data over a 20 mm mat depth horizon, with the surface layer possessing over 200 BGCs and containing the highest relative abundance of BGCs of all mat layers. Terpene and bacteriocin BGCs were highly represented and their natural products are proposed to have important roles in ecosystem function in these mat systems. Interestingly, potentially novel BGCs were detected from Heimdallarchaeota and Lokiarchaeota, two evolutionarily significant archaeal phyla not previously known to possess BGCs. This study provides new insights into how secondary metabolites from BGCs may enable diverse microbial mat communities to adapt to extreme environments.

## Introduction

Microorganisms in the environment can produce a wide range of secondary metabolites with diverse chemical structures (Katz and Baltz, 2016). The diversity in chemical structures enable these natural products to perform a variety of functions. Many natural products today are important components of human medicine and industry by acting as antibacterials, antitumor agents, insecticides, and many more (Schwecke et al., 1995; Brautaset et al., 2000; Waldron et al., 2001; Liu et al., 2016). Approximately 70% of the anti-infective drugs used in human medicine have been derived from natural products (Newman and Cragg, 2016). Secondary metabolites are not directly associated with the growth of microorganisms but are known to provide benefits to the host producer by acting as growth inhibitors against rival bacteria, metal transporting agents, and quorum sensing molecules (Demain and Fang, 2000).

Biosynthetic gene clusters (BGCs) are a locally clustered group of two or more genes that together encode a biosynthetic pathway for the production of a secondary metabolite (Medema et al., 2015). Different structural classes of BGCs exist including non-ribosomal peptide synthetases (NRPS), polyketide synthases (PKS), terpenes, and bacteriocins. NRPS and PKS are popular targets for natural product discovery as they are known to synthesize a diversity of antibiotics and immunosuppressants with enormous pharmaceutical potential (Schwecke et al., 1995; Tillett et al., 2000; Wang et al., 2014). Condensation (C) domains from NRPS clusters are functionally active protein sequences that catalyze the amide bond formation, an important step in peptide elongation (Bloudoff and Schmeing, 2017). Similarly, ketosynthase (KS) domains catalyze the condensation reaction in PKS clusters (Wang et al., 2014). These domains are suitable targets in genomic analysis as they are highly conserved and can be used to distinguish between different NRPS/PKS natural product pathways (Ziemert et al., 2012).

Previous work to uncover biosynthetic systems in uncultivated microorganisms have relied on cloning environmental DNA into host organisms to screen for function (Rondon et al., 2000). Other studies have utilized degenerate PCR primers to search for BGCs without the need for cloning (Charlop-Powers et al., 2014, 2015). However, primers become increasingly difficult to design for large gene families and can fail to amplify novel sequences of interest (Linhart and Shamir, 2005). Using a metagenomics workflow, we can reconstruct near-complete genomes *de novo*. This enables for potentially novel BGCs to be identified in both culturable and unculturable microorganisms as well as infer phylogenies and their ecological contribution in specific environments. antiSMASH is an *in silico* pipeline offering detection and analysis of many BGC types (Blin et al., 2017b). This pipeline was previously validated against a database of 473 verified BGCs with a high reported accuracy of 97.7% (Medema et al., 2011). BGC detection pipelines have significantly advanced our understanding of a range of ecologically and evolutionarily significant environments where natural product discovery has substantial potential, however, one environment where there is a knowledge gap in terms of BGCs are microbial mats.

Microbial mats are organo-sedimentary multilayered ecosystems that are host to a diverse community of microorganisms (Prieto-Barajas et al., 2018). Recent work has demonstrated microbial mats emerged as far back as 3.7 billion years (Nutman et al., 2016), and thus these systems hold substantial evolutionary significance. Shark Bay in Western Australia has extensive microbial mat systems, and the microbial communities have been studied in detail at the taxonomic and functional levels using a range of methods (Leuko et al., 2007; Allen et al., 2008; Wong et al., 2017, 2018; White et al., 2018, 2020). Mats located in Shark Bay are constantly exposed to high UV radiation, hypersalinity, and desiccation stresses (Burns et al., 2009; Wong et al., 2015). Recent metagenomic studies have shown microbial networks and feedback loops are key in maintaining ecosystem function and stability in modern stromatolites (Prieto-Barajas et al., 2018), and it has been suggested the Shark Bay systems harbor microorganisms and functional genes that might have been prevalent in ancient microbial mats (Wong et al., 2018). Our data has shown that metabolic specialization may allow switching metabolic pathways at specific depth horizons in microbial mats, and these findings are part of a changing paradigm regarding the capability of microbial networks to function under seemingly inhospitable conditions. However, to date few studies have delineated the presence and potential significance of BGCs in microbial mats, despite the potential for biotechnological applications in these systems (Prieto-Barajas et al., 2018). The present study thus aimed to uncover the diversity of BGCs from microbial mats in Shark Bay, search for potential BGCs in understudied microorganisms, and identify the ecological functions of secondary metabolites in this particular ecosystem, via in-depth analyses of metagenome assembled genomes (MAGs) using antiSMASH and NaPDoS pipelines.

## Materials and Methods

### Microbial Mat Sampling, Metagenomic Sequencing, and Data Availability

Sampling and metagenomic sequencing of Shark Bay microbial mats was undertaken previously (Wong et al., 2015, 2018). Briefly, triplicate mat samples were aseptically taken in April 2013 from Nilemah (26°27′336′′S, 114°05.762′′E), sliced into ten 2 mm layers, and total community DNA extracted. After quality control and library preparation, shotgun sequencing on an Illumina NextSeq 500 platform was performed in duplicate on each layer.

### Quality Control, Assembly, and Binning

The quality of the reads was assessed with FastQC version 0.11.6 and low-quality bases (per base sequence quality <28) were removed with Trimmomatic version 0.36 (Bolger et al., 2014; Andrews, 2020). All sequencing files from the ten layers were co-assembled (minimum kmer 27, with incremental kmer of 10) with Megahit version 1.1.1 as described (Li et al., 2015; Stewart et al., 2018). After contigs assembly, only contigs with length more than 2000 bp were retained to avoid ambiguous gene annotation and binning errors from shorter contigs. BWA-MEM version 0.7.7 was used to map reads back to the assembled contigs (Li and Durbin, 2009). MAGs were constructed with MetaBAT2 version 2.12.1 (Kang et al., 2015). The completeness and contamination values of each MAG was assessed by CheckM version 1.0.12 (Parks et al., 2015). High-quality MAGs (>90% completeness and <5% contamination) were then selected for taxonomic assignment and BGC detection as recommended in the antiSMASH pipeline (Blin et al., 2017a).

### Taxonomic Assignment and Phylogenetics

Taxonomic assignment to each high-quality MAG was done using GTDB-tk version 0.2.1. Furthermore, fifteen ribosomal proteins from the large and small subunit (rpL2, rpL3, rpL4, rpL5, rpL6, rpL14, rpL15, rpL18, rpL22, rpL24, rpS3, rpS8, rpS10, rpS17, and rpS19) were then generated for all high-quality MAGs using Phylosift version 1.0.1 (Darling et al., 2014). The fifteen ribosomal proteins listed above are known to be ubiquitous in bacteria and archaea and hence suitable for building a phylogenetic tree to represent various bacterial and archaeal groups in an environmental (Darling et al., 2014). Sequences for each ribosomal protein were merged together and then aligned using MAFFT version 7.310, a multiple sequence alignment program (Katoh et al., 2002). Any gaps in the aligned sequences were removed using BMGE version 1.12 (Criscuolo and Gribaldo, 2010). After removing gaps in the aligned sequences, all aligned ribosomal protein sequences were merged to create a single file. A phylogenetic tree was constructed by maximum likelihood method based on 1000 bootstrap replications, via IQ-TREE version 1.6.9 (Nguyen et al., 2014). The tree was visualized and annotated using iTOL version 3.5.3 (Letunic and Bork, 2019).

### Detection of BGCs in Shark Bay Mat Metagenomes

The overall file size of each metagenome layer was reduced to improve processing and detection of BGCs over 1 kb which was previously recommended (Blin et al., 2017a). To achieve this, contigs less than 1 kb in length were removed from each metagenome file. Annotation and labeling of relevant genomic features on contigs were done using Prokka version 1.13 (Seemann, 2014). Prediction of BGCs in mat metagenomes was performed using antiSMASH version 4.2.0 with minimum contig length at 1000 bp and the minimal detection option selected so that only BGCs are detected (Blin et al., 2017b). Predicted BGCs in each metagenome were compiled and the top ten highly abundant BGCs in each mat layer were represented in a bar chart generated using Rstudio with ggplot2 and RColorBrewer packages.

### Comparison of Bacteriocin Clusters Between Microbial Mat Layers

Bacteriocin gene clusters detected in the top (0–2 mm), middle (8–10 mm), and bottom microbial mat layer (18–20 mm) were selected for further analysis due to their abundance and distribution throughout each mat layer. Ninety-five protein sequences from bacteriocin clusters were extracted from antiSMASH output and were blasted against the non-redundant protein sequences database with NCBI BLASTP (cut-off *e*-value > 10^–6^, amino acid identity cut-off <30%, and bit-score cut-off <50). The top three hits for each protein sequence were represented in Supplementary Table S5.

### Detection and Analysis of BGCs in MAGs

From the group of high-quality MAGs, fifteen candidate MAGs of different assigned phyla were selected for BGC detection (Supplementary Table S1). The MAGs were passed through antiSMASH version 4.2.0 with minimum contig length at 1000 bp and all analysis options selected except for the ClusterFinder algorithm. The KnownClusterBlast algorithm was also selected in order to compare predicted BGCs to the Minimum Information about a Biosynthetic Gene cluster (MIBIG) database (Medema et al., 2015). The MIBIG database contains BGC entries that have been experimentally characterized and can be used to infer natural products encoded by detected BGCs. A heatmap representing MAGs and the number of BGCs detected was constructed using Rstudio with ggplot2 and reshape2 packages.

### Analysis of C and KS Domains From NRPS and PKS Clusters

To identify potential natural product pathways from NRPS and PKS gene clusters, the NaPDoS pipeline was used to compare C and KS domain sequences to a domain database of previously characterized natural products (Ziemert et al., 2012). The C and KS domains from NRPS and PKS detected in the fifteen candidate MAGs were extracted from antiSMASH output and subsequently analyzed using the NaPDoS web server with default settings. A phylogenetic tree was constructed by maximum likelihood using NaPDoS for all C domains. A separate phylogenetic tree for all KS domains was also constructed by maximum likelihood using NaPDoS. The trees were then visualized and annotated using iToL.

## Results and Discussion

### Metagenomic Data Summary of Shark Bay Microbial Mats

A total of 761 MAGs were recovered from the binning process. Of these, 160 MAGs were classified as high-quality drafts (>90% completeness and <5% contamination) according to recently established standards (Bowers et al., 2017).

### Microbial Mat Community Composition

**FIGURE 1:**
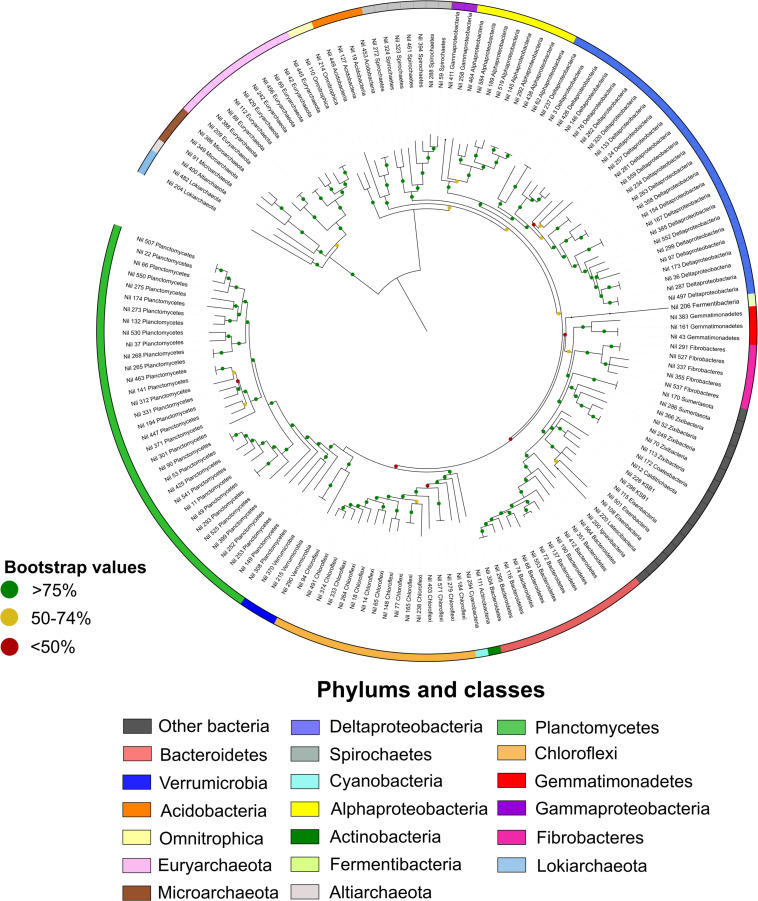
Maximum likelihood phylogenetic tree of Shark Bay microbial mat microorganisms. In total 160 high-quality MAGs were assigned color coded taxonomies. The outer ring represented the distribution of phyla in Shark Bay microbial mats. Proteobacteria were divided into Alphaproteobacteria and Deltaproteobacteria and bootstrap values were indicated at each node in the tree. Planctomycetes and Deltaproteobacteria represented a large proportion of high-quality MAGs.

A total of 27 phyla were identified based on phylogenetic analyses of the mat metagenomes (Figure 1). Planctomycetes represented 22% of MAGs, followed by Deltaproteobacteria (16%), and Chloroflexi (11%). This is in support of previous metagenomic studies and total 16S rRNA community data have shown that microbial mats are dominated by Deltaproteobacteria, Cyanobacteria, Planctomycetes, Spirochaetes, Chloroflexi, and Bacteroidetes (Wong et al., 2015, 2018).

Microbial dark matter – the vast array of microorganisms in the environment with no cultured representatives – MAGs such as “Candidatus Zixibacteria,” “Candidatus Eisenbacteria,” “Candidatus Sumerlaeota,” and “Candidatus KSB1” were also uncovered here (Figure 1 and Supplementary Table S1). The metabolism and functions of these latter bacterial groups are poorly understood but were suggested to have minimal genome capacities and require hosts for symbiotic lifestyles (Castelle et al., 2018). Two MAGs assigned as Lokiarchaeota (90.89% completeness and 9.76% contamination) and Heimdallarchaeota (95.33% completeness and 9.81% contamination) were also uncovered and assessed for potential BGCs as the secondary metabolism of Asgard archaea remains largely unexplored. The metabolic functions of Lokiarchaeota have recently been proposed in a number of studies (Sousa et al., 2016; Wong et al., 2018), however, prior to the present study it has not been shown whether this phylum possess the capacity for secondary metabolism.

### BGC Abundance Comparison in Different Microbial Mat Layers

**FIGURE 2:**
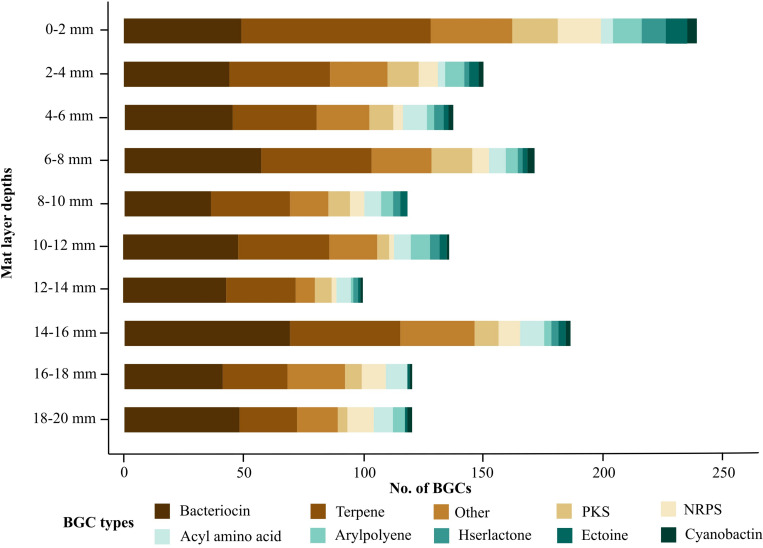
Abundance of the top ten BGC types at each microbial mat layer. Biosynthetic gene clusters were detected on contigs greater than 1 kb. BGC counts were performed at each 2 mm depth from the surface (0–2 mm) to the bottom layer (18–20 mm). Bacteriocin and terpene clusters were highly represented throughout each microbial mat layer.

The metagenomes from each microbial mat layer depth were analyzed with antiSMASH to compare their biosynthetic potential. A total of 1477 BGCs were detected with each layer having at least over 100 BGCs (Figure 2). Deeper mat layers were relatively less enriched with BGCs although the overall types of BGCs detected in each layer were not very different. A similar trend was also observed in a recent soil study where genomes from shallow samples were more enriched with BGCs compared to genomes from deeper samples (Sharrar et al., 2020). Natural product discovery efforts have mainly focused on marine and soil environments due to their rich biodiversity and biosynthetic potential. The BGC abundance observed in our dataset were found to be as high as those found in soil ecosystems and more enriched compared to a metagenomics lake study (Crits-Christoph et al., 2018; Cuadrat et al., 2018). We report the distribution of BGCs in Shark Bay microbial mats comparable with BGCs recovered from soil and water ecosystems thus supporting Shark Bay microbial mats as a possible new source of untapped biosynthetic potential.

The surface layer had over 200 BGCs and contained the highest amount of BGCs of all mat layers (Figure 2). A previous 16S rRNA survey of Shark Bay microbial mats revealed the surface layer being dominated and over-represented by Alpha-, Gammaproteobacteria, Bacteroidetes, and Cyanobacteria (Wong et al., 2015). A recent study also found BGCs from Gammaproteobacteria were enriched in shallow sections of soil (Sharrar et al., 2020). Gammaproteobacteria and Cyanobacteria are known prolific secondary metabolite producers and Bacteroidetes have previously been reported to encode for a range of bacteriocins thus supporting the relatively high count of BGCs at the surface layer (Wang et al., 2014; Lopetuso et al., 2019; Reddy et al., 2020). The high distribution of BGCs at the microbial mat surface may also be attributed to microbial adaptations against harsh environmental conditions due to the surface layer being directly exposed to high UV radiation and desiccation stress (Wong et al., 2018). In this study, terpene gene clusters represented up to 33% of the BGCs in the microbial mat surface (Figure 2). Terpene gene clusters are responsible for producing a wide range of functionally diverse secondary metabolites such as carotenoids providing colorful pigments and geosmin, an odorous metabolite produced mainly by Cyanobacteria (Pattanaik and Lindberg, 2015). A function of geosmin has been associated with the removal of excess metabolites when microorganisms are subjected to stress events (Watson, 2003), and a similar role could be occurring in microbial mats. Photosynthetic Cyanobacteria have previously been observed to synthesize several carotenoids and syctonemin, a secondary metabolite synthesized in response to UV exposure (Balskus et al., 2011; Galasso et al., 2017). Photosynthetic Cyanobacteria in Shark Bay microbial mats have previously been shown to produce syctonemin (D’Agostino et al., 2019), and the high representation of Cyanobacteria at the surface layer supports the relatively high number of surface terpene gene clusters observed in this study (Burns et al., 2005; Fisher et al., 2018). We hypothesized that high UV exposure at the surface layer of Shark Bay microbial mats have led to adaptations in Cyanobacteria encoding for additional terpene gene clusters. These gene clusters can produce a range of UV protective secondary metabolites which can absorb incoming UV radiation and subsequently shield the wider microbial mat community from harmful UV radiation.

A previous clone library survey of Shark Bay microbial mats first proposed the genetic potential for secondary metabolism in this ecosystem due to the identification of genes from NRPS and PKS encoding for enzymes responsible for the production hepatotoxins and antibiotics (Burns et al., 2005). However, the identification of gene clusters from this study was limited by the specificity of primers which allowed for only PKS/NRPS fragments to be amplified and cloned. Cultured strains of Cyanobacteria were also required in this study thus greatly limiting the search for BGCs. In comparison to the present study, we greatly expand the classes of BGCs detected in Shark Bay microbial mats and provide a greater resolution of BGC abundance and diversity present in this ecosystem.

There were a total of 77 NRPS and 101 PKS clusters detected from all layers combined, likely indicating microorganisms in all layers may encode for a range of secondary metabolites conferring benefits to the host or microbial community (Figure 2). In comparison, genomes of *Bacillus paralicheniformis* recovered from the hypersaline microbial mats of Rabigh Harbor Lagoon by the Red Sea in Saudi Arabia were found to be enriched with 480 BGCs and indicate the potential adaptations undertaken by microorganisms to survive the relatively hot and hypersaline ecosystems (Othoum et al., 2018). Here we report a relatively high abundance of NRPS and PKS clusters which may be attributed by adaptations undertaken by the microbial community to survive the extreme conditions in Shark Bay.

Bacteriocin and terpene gene clusters dominated in all layers and contributed over 20% of the total BGCs detected (Figure 2). Other studies have also observed similar trends of bacteriocin and terpene gene clusters dominating environments such as lakes, agricultural groundwater, and soil (Ludington et al., 2017; Cuadrat et al., 2018; Sharrar et al., 2020). The abundance of bacteriocins and terpenes in different environments suggests the secondary metabolites encoded by these gene clusters may play important roles in the survival and adaptation of the microbial community. In the present study, we found terpene gene clusters still represented approximately 20% of the BGCs recovered from deeper microbial mat layers despite exposure to significantly lower levels of light and UV radiation (Figure 2). Notably, some Cyanobacteria species and carotenoids encoded by terpene gene clusters were previously reported to be abundant in deeper layers of Shark Bay microbial mats hence supporting photosynthetic bacteria undergoing adaptations to lower light conditions (Wong et al., 2015; Fisher et al., 2018). Here we hypothesize that the proportion of terpene gene clusters recovered from deeper layers of microbial mats may be contributed by a population of Cyanobacteria adapted to low light conditions. Furthermore, bacteriocin gene clusters were also widely distributed throughout microbial mat depths thus providing an opportunity to assess the protein sequences of these bacteriocin gene clusters for potential novelty.

### Bacteriocin Novelty in Microbial Mats

From the 95 protein sequences analyzed, a nitrogen-fixation protein (Nif11) represented 20% of matches (Supplementary Table S5). The Nif11 protein was previously reported to be associated with photosynthetic Cyanobacteria and Proteobacteria (Jacobson et al., 1989; Kaneko et al., 2001). While Nif11 plays a role in the nitrogen cycle, the identification of this protein within bacteriocin gene clusters is unusual and may suggest this protein could be repurposed for secondary metabolism. One particular study also reported evidence of Nif11 serving as natural product precursors (Haft et al., 2010). Previous studies have reported the widespread distribution of bacteriocin and their capacity to function as colonizing peptides, signaling molecules, or as killing peptide molecules (Dobson et al., 2012). Here we highlight the abundance and potential novelty of bacteriocin clusters in Shark Bay microbial mats which were previously not reported in this ecosystem. Future transcriptomic work will help determine expression levels of bacteriocin and indicate if fluctuating temperatures and hypersaline conditions may induce higher bacteriocin production. Notably, hypothetical proteins and domains of unknown function were the top hits in approximately 38% of sequences (Supplementary Table S5), inferring potentially novel bacteriocins present. With bacteriocin clusters dominating most microbial mat layers, we also report the potential for novel bacteriocin clusters. Unculturable microorganisms may also be contributing to the largely unknown portion of putative BGC sequences yet to be annotated and characterized (Bernard et al., 2018).

### Detection of BGCs in MAGs With Assigned Taxonomies

**FIGURE 3:**
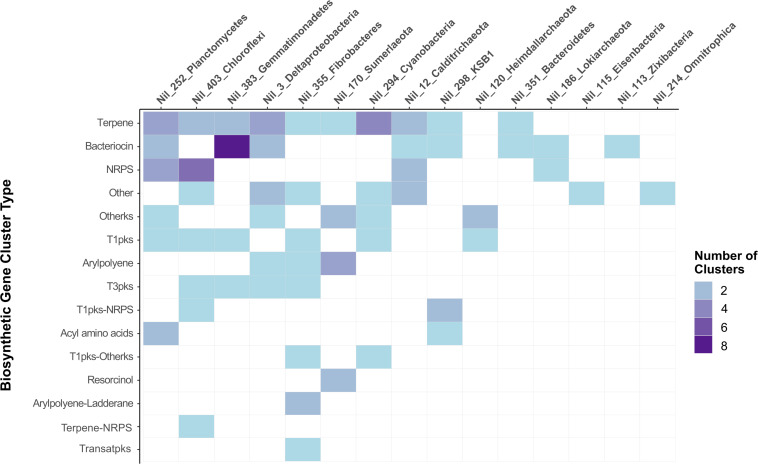
BGCs detected by antiSMASH in fifteen MAGs of different assigned phyla. MAGs are arranged left to right by highest number of BGCs while gene clusters were arranged top to bottom by abundance of BGC type. The most abundant BGCs were terpenes followed by bacteriocin and NRPS clusters. The greatest number of BGCs were found in Planctomycetes, followed by Chloroflexi and Gemmatimonadetes.

Biosynthetic gene cluster analysis was carried out on two archaeal MAGs and thirteen bacterial MAGs to link their taxonomies with their putative secondary metabolism (Supplementary Table S2). Terpene and bacteriocin BGCs were most abundant and occurred in ten and eight phyla, respectively (Figure 3). The greatest number of BGCs were detected in Planctomycetes (Nil_252), Chloroflexi (Nil_403), and Gemmatimonadetes (Nil_383). The Planctomycetes and Chloroflexi phyla are commonly found in soil and marine sponges and share characteristics with highly bioactive Actinobacteria such as large genomes and complex life cycles (Graça et al., 2016). Studies on Chloroflexi inhabiting marine sponges have detected novel NRPS and PKS as well as terpene clusters in their genomes suggesting their suitability for natural product discovery (Siegl and Hentschel, 2010; Bayer et al., 2018). An antiSMASH survey of the Planctomycetes genome also recovered numerous ladderane, bacteriocin, terpene, and fatty acid BGCs (Ludington et al., 2017). Similarly, we found several NRPS, PKS and terpene gene clusters in Planctomycetes and Chloroflexi which further highlights the high biosynthetic potential of these phyla (Figure 3).

Gemmatimonadetes represents one of many poorly understood bacterial phyla with only a few strains successfully cultured (Zeng et al., 2016). While commonly found in soil, few reference genomes are available and previous studies found evidence of some BGCs present in their genome (Wang et al., 2014; Crits-Christoph et al., 2018). From the twelve BGCs detected in Gemmatimonadetes, eight bacteriocins were detected suggesting this phylum may contain a greater biosynthetic capacity than previously thought (Figure 3). Similarly, a recent metagenomics soil study described bacteriocins being the prominent gene cluster detected in the genomes of Gemmatimonadetes (Sharrar et al., 2020). We report the prevalence of bacteriocin clusters in the genome of Gemmatimonadetes recovered from hypersaline microbial mats which appear to share similar biosynthetic capacities seen in the genomes of Gemmatimonadetes inhabiting soil environments.

Microbial phyla affiliated with microbial dark matter possessing biosynthetic potential include “Candidatus KSB1” and “Candidatus Sumerlaeota.” Terpene and PKS clusters were detected in these phyla and their KS domains were subjected to further analysis in NaPDoS (Figure 3). To our knowledge, their secondary metabolisms have not been previously reported. Only one BGC was detected in “Candidatus Zixibacteria,” “Candidatus Omnitrophica,” and “Candidatus Eisenbacteria.” This is in contrast with a recent study which found a “Candidatus Eisenbacteria” genome encoding for NRPS and PKS domains which are comparable with some Actinobacteria genomes which are well-known for their incredible biosynthetic capacity (Wohlleben et al., 2016). In another study, the genomes of “Candidatus Omnitrophica” revealed terpene clusters involved in the non-mevalonate pathway (Ludington et al., 2017). Due to the lack of BGCs detected in these phyla, more work may be needed to recover more genomes from Shark Bay microbial mats before assessing the biosynthetic potential of these phyla. Nonetheless, we found several genomes containing NRPS or PKS including Cyanobacteria, Planctomycetes, Chloroflexi, Fibrobacteres, “Candidatus Sumerlaeota,” “Candidatus KSB1,” Lokiarchaeota and Heimdallarchaeota (Figure 3). The C and KS domains were extracted from these gene clusters and were subjected to NaPDoS analysis to link NRPS and PKS systems to potential natural products.

### Prediction of NRPS and PKS Products Encoded by Microbial Mat Microorganisms

The threshold for determining functional gene novelty remains debatable. According to NaPDoS, sequences with hits below 85% may indicate the domain of interest may contribute to natural products that have not yet been characterized (Ziemert et al., 2012). We found an overwhelming majority of C and KS domain sequences having sequence identity scores well below 85% to their predicted natural product (Supplementary Tables S2, S3). It was previously shown that shotgun metagenomics may lead to fragments of NRPS/PKS gene clusters which may account for the large number of low identity scores (Meleshko et al., 2019). On the contrary, scores below the threshold may imply potential novelty in NRPS and PKS products, although further studies would be required to assess the extent of this novelty.

**FIGURE 4:**
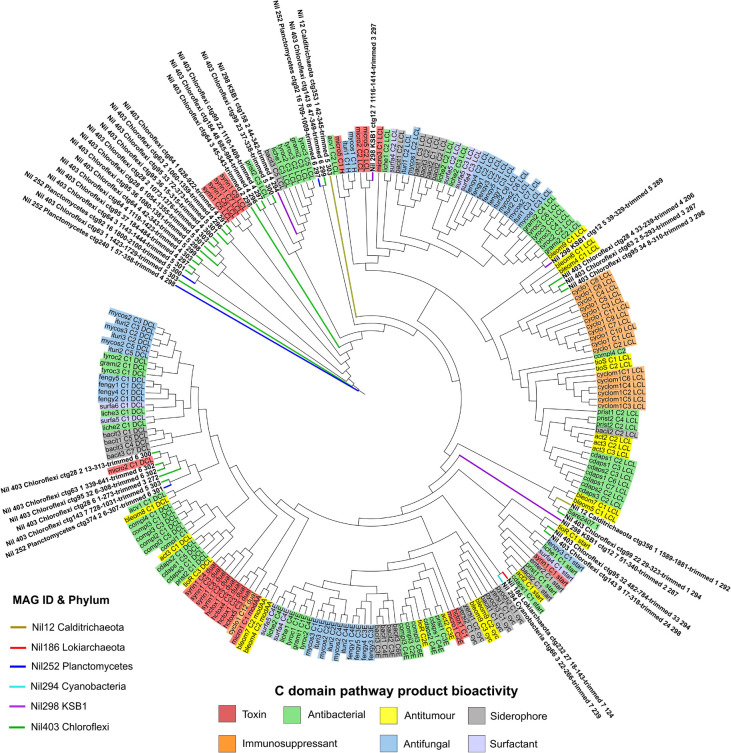
Phylogenetic tree of ketosynthase domains by maximum likelihood method against the NaPDoS domain database. Natural products are represented on the outer ring and are shaded according to their bioactivity. Domains from MAGs are color coded and their branches are highlighted on the tree. Several KS domains from Deltaproteobacteria, Spirochaetes and Chloroflexi aligned closely to antitumor compounds. KS domains from Heimdallarchaeota were distant from any natural products in the database with PUFA compounds being the nearest. Several KS domains from Fibrobacteres, Planctomycetes, and Sumerlaeota aligned near poly-unsaturated fatty acid (PUFA) compounds.

Several KS domain sequences from Planctomycetes and Cyanobacteria aligned with poly-unsaturated fatty acid (PUFA) compounds from *Azotobacter* and *Shewanella* genus with up to 54 and 64% identity, respectively (Figure 4 and Supplementary Table S3). PUFA compounds are well-known for their photoprotective properties against harsh UV radiation which damage the cell wall and membranes of microorganisms. A previous study employing bioinformatics and chemical analyses on the Shark Bay microbial mat community revealed a higher proportion of UV screening compounds present in the upper layer of microbial mats and found the abundance of Cyanobacteria influences the occurrence of scytonemin, a secondary metabolite with photoprotective properties (D’Agostino et al., 2019; Pathak et al., 2019). We report Planctomycetes and Cyanobacteria as the dominating phyla and main producers of PUFA on the surface layer of Shark Bay microbial mats which provide the microbial community shielding against high UV radiation.

Surprisingly, no KS domains from Cyanobacteria’s PKS cluster matched to any toxin products despite numerous studies on microcystins which are a major class of toxins synthesized by Cyanobacteria (Figure 4; Bláha et al., 2009). In contrast, a metagenomics study on Cyanobacteria dominated microbial mats from the Eel River Network in California identified the natural product anatoxin-a from numerous Cyanobacterial genomes (Bouma-Gregson et al., 2019). We report the absence of Cyanobacteria gene clusters encoding for toxins in our dataset. Recovery of additional Cyanobacterial genomes for analysis would provide stronger support for these observations.

**FIGURE 5:**
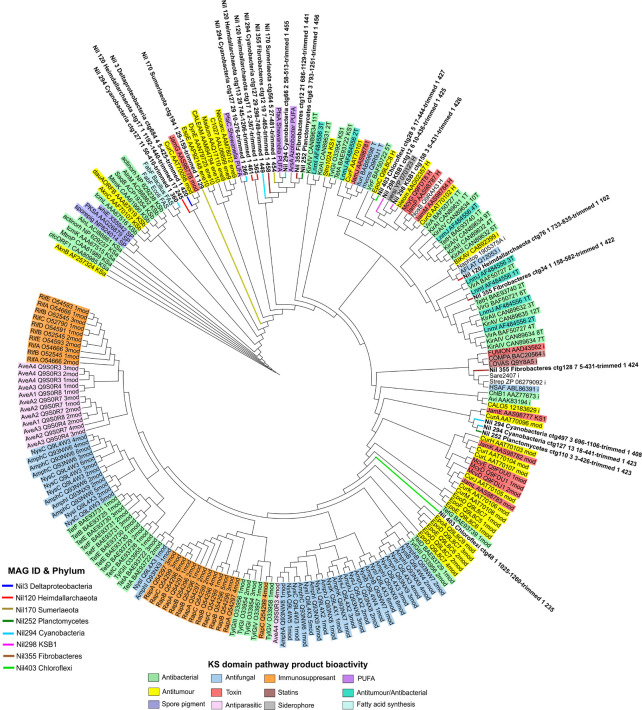
Phylogenetic tree of condensation domains by maximum likelihood method against the NaPDoS domain database. Natural products are represented on the outer ring and are shaded according to their bioactivity. Domains from MAGs are color coded and their branches were highlighted on the tree. Various C domains aligned most closely to pathways encoding for toxins and antibacterial compounds. C domains from Lokiarchaeota and Cyanobacteria were distant from natural products in the database.

The secondary metabolism of the Chloroflexi phylum is generally understudied with recent studies only beginning to uncover their secondary metabolism (Sharrar et al., 2020). Initial surveys of Chloroflexi’s genome reveal the presence of some NRPS and PKS clusters (Siegl and Hentschel, 2010; Wang et al., 2014; Bayer et al., 2018). In the present study, we found 17 of 28 C domains from Chloroflexi aligned closely with syringomycin (Figure 5 and Supplementary Table S4). Syringomycin is a phytotoxin compound belonging to the same family of lipopeptides which exhibit antimicrobial and biosurfactant properties (Raaijmakers et al., 2010). Biosurfactants are functionally versatile natural products sought after in medicine and biotechnology. Of ecological significance, biosurfactants enable microorganisms to grow on water-immiscible substrates by reducing the surface tension between interfaces thus facilitating greater nutrient uptake (Desai and Banat, 1997). This is especially relevant in microbial mats as a complex microbial community develops at the interfaces of water and biosedimentary minerals (Allen et al., 2009). This may also contribute to Chloroflexi being the most abundant bacteria in Shark Bay microbial mats given the oligotrophic environment (Wong et al., 2015). Here we expand on the secondary metabolism of Chloroflexi and report a potential NRPS cluster from Chloroflexi which may encode for a novel syringomycin-like product with potential ecological roles in enabling greater nutrient uptake and metabolism in microbial communities.

### Analysis of KS and C Domains From Understudied Phyla

Ketosynthase domains from Heimdallarchaeota showed alignment to aflatoxin, an antifungal compound (Figure 4). PUFA domains were also the nearest hits according to NaPDoS (Supplementary Table S3). It is not clear if there is an association with Heimdallarchaeota and antifungal secondary metabolism as this was not previously reported. According to BLASTP, acetyltransferase, and beta-ketoacyl synthase specific to Heimdallarachaeota had 72% and 81% identity to KS domains from our Heimdallarchaeota MAG, respectively (Supplementary Table S3). This implies the presence of PKS clusters in the Heimdallarchaeota genome as acetyltransferase and beta-ketoacyl synthase are key biosynthetic components (Cummings et al., 2014). This study reports a potentially novel PKS cluster in Heimdallarchaeota’s genome which may encode for a product sharing some similarity to PUFA or antifungal compounds.

Phylogenetic analyses revealed that the C domain from Lokiarchaeota did not align with any natural product although mycosubtilin, an antifungal compound is reported as the nearest hit at 28% similarity (Figure 5 and Supplementary Table S4). A recent study found evidence of Asgard archaeal genomes possessing transcriptional regulators which are components of putative archaeal offense systems and toxin-antitoxin systems (Makarova et al., 2019). This seems to suggest that Asgard archaeal genomes have the capacity to encode for complex systems. While inconclusive at this stage, the lack of similarity to a natural product may suggest that Lokiarchaeota’s genome may hold an unusually novel NRPS-like machinery not yet characterized. Here we describe the possibility of secondary metabolism in Lokiarchaeota which has not been previously shown.

A KS domain from “Candidatus KSB1” aligned closely with yersiniabactin, a siderophore molecule involved in transporting iron across cell membranes (Figure 4; Perry and Fetherston, 2011). Siderophores can also bind to a variety of metals to supply essential elements to microorganisms (Ahmed and Holmström, 2014). Iron is an essential element for the growth of most microorganisms by acting as a catalyst in enzymatic processes such as oxygen metabolism and for stabilizing the polysaccharide matrix during biofilm formation (Ahmed and Holmström, 2014). The identification of a KS domain in KSB1 with similarity to a siderophore compound suggests this phylum may play an important role in scavenging metals and biofilm formation in microbial mats. Observations at this stage are speculative and will require future investigation to validate the presence of these PKS clusters in this candidate phylum.

Ketosynthase domains from Fibrobacteres and “Candidatus Sumerlaeota” aligned with PUFA from the *Shewanella* and *Azotobacter* genus with 54% and 53% similarity, respectively (Figure 4 and Supplementary Table S3). PUFA are physiologically important compounds that take part in facilitating the function of individual membrane proteins in bacteria (Yoshida et al., 2016). They have been observed to provide bacteria with membrane-shielding function against reactive oxygen species (ROS; Nishida et al., 2006). Consequently, high UV exposure and hypersaline conditions in the Shark Bay environment can induce greater amounts of ROS such as peroxide and hydroxyl radicals which are toxic to microbial communities (Wong et al., 2018). Due to the low similarity score, we propose Fibrobacteres and Sumerlaeota may encode potentially novel PUFA compounds that may serve as a defense mechanism against elevated levels of ROS in microbial mats. This study also expands our knowledge of the understudied and unculturable phyla Fibrobacteres and Sumerlaeota which were previously not known to encode for PUFA.

## Conclusion

Microorganisms living in Shark Bay microbial mats are exposed to harsh environmental conditions that require numerous survival responses. Microbial mat microorganisms may encode a wealth of natural products that serve various survival benefits and have been exploited by society in the field of medicine and biotechnology. Using a metagenomics approach, this study reports the abundance and variety of BGCs recovered from microbial mat microorganisms with the potential for some novel BGCs encoding for new natural products in deep-branching archaea and microbial dark matter. Microbial mat microorganisms that are exposed to harsh environmental stressors. This study has increased our understanding of microbial adaptation in the extreme environment of Shark Bay microbial mats and provides a framework for future studies mapping secondary metabolism pathways of microbial mat microorganisms and their potential to encode for new natural products of interest.

## Data Availability Statement

The datasets presented in this study can be found in online repositories. The names of the repository/repositories and accession number(s) can be found at: https://www.mg-rast.org/linkin.cgi?project=mgp81948, 4762868.3 to 4762965.3.

## Author Contributions

RC performed antiSMASH analyses on metagenomic data and drafted the manuscript. HW, GK, and FM conducted assembly, binning, and other specific data analyses. NB and BF provided detailed data analyses and interpretation. BB designed and led the study, and wrote specific manuscript sections. All authors edited the final manuscript.

## Conflict of Interest

The authors declare that the research was conducted in the absence of any commercial or financial relationships that could be construed as a potential conflict of interest.
